# Impact of age, sex, and thyroid autoimmunity on the association between selenium intake and type 2 diabetes mellitus

**DOI:** 10.1186/s12889-024-18225-2

**Published:** 2024-03-08

**Authors:** Xiao-Man Ma, Ke-Xuan Li, Zi-Qiu Chen, Cai-Mei Wu, Wan-Zhe Liao, Xu-Guang Guo

**Affiliations:** 1 Department of Clinical Laboratory Medicine, Guangdong Provincial Key Laboratory of Major Obstetric Diseases; Guangdong Provincial Clinical Research Center for Obstetrics and Gynecology; The Third Affiliated Hospital of Guangzhou Medical University, Guangzhou, 510150 China; 2 Department of Clinical Medicine, The Third Clinical School of Guangzhou Medical University, Guangzhou, 511436 China; 3 Guangzhou Key Laboratory for Clinical Rapid Diagnosis and Early Warning of Infectious Diseases, King Med School of Laboratory Medicine, Guangzhou Medical University, Guangzhou, 510000 China

**Keywords:** Selenium intake, T2DM, Thyroid autoimmunity, Threshold effect, NHANES

## Abstract

**Background:**

The association between dietary selenium(Se) intake and type 2 diabetes mellitus (T2DM) remains controversial. The present study aimed to investigate this association using data from the National Health and Nutrition Examination Survey (NHANES) database for the years 2007–2012.

**Methods:**

Three thousand seventy three individuals aged 20 years and above were eligible for inclusion in this cross-sectional study. The average age of the participants was 50.74 years and the proportions of males and females were nearly equal (49.12% vs. 50.88%). The odds ratios (OR) of the association between dietary Se intake (log2-transformed) and T2DM were examined through the multivariate logistic regression model. Subgroup analyses were conducted based on age, sex, and thyroid autoimmunity to assess the potential impact of these variables on the relationship. Fitted smoothing curves and threshold effect analysis were conducted to describe the nonlinear relationship.

**Results:**

In the fully adjusted model, a significant positive association between Se intake and T2DM was observed (OR = 1.49, 95% CI: 1.16, 1.90, *p* = 0.0017). After stratifying the data by age, sex, and thyroid autoimmunity, a significant positive association between Se intake and T2DM was observed in individuals under 65 years of age, males, and those with negative thyroid autoimmunity. A two-segment linear regression model was analyzed for sex stratification, revealing a threshold effect in males with an inflection point of 90.51 μg, and an inverted U-shaped relationship in females with an inflection point of 109.90 μg, respectively.

**Conclusions:**

The present study found a positive relationship between Se intake and the prevalence of T2DM. This association is particularly significant in younger individuals, males, and those with negative thyroid autoimmunity. Our results should be validated in future large prospective studies in different populations.

**Supplementary Information:**

The online version contains supplementary material available at 10.1186/s12889-024-18225-2.

## Background

Type 2 diabetes mellitus(T2DM) is a rapidly growing global health crisis in the twenty-first century, which results in various cardiovascular diseases, reduced quality of life, and substantial medical expenses [1, 2]. According to the latest data from the International Diabetes Federation, in 2021, over 500 million people worldwide were affected by T2DM, accounting for more than 10.5% of the global adult population [2]. Additionally, it is projected that the number of individuals affected by T2DM will surpass 780 million by 2045 [2]. While there is a strong genetic basis for susceptibility to T2DM in individuals, epidemiological research evidence suggests that many cases of T2DM can be prevented by lifestyle modifications [3]. Hence, it is crucial to comprehend the risk factors associated with T2DM and implement effective treatment measures to effectively manage the condition. The primary risk factors for T2DM include being overweight or obese, dietary choices, level of physical activity, smoking, and alcohol consumption. A review indicates that there are correlations between the risk of developing T2DM and the intake of specific nutrients, food groups, and overall dietary patterns [3]. Extensive researches have been conducted to examine the connections between various nutritional factors, such as iron, docosahexaenoic acid (DHA) or eicosapentaenoic acid (EPA), vegetable fiber, fruit fiber, α-Linolenic acid, magnesium, and vitamin D, and the development of T2DM [4–7].

Many studies have shown an increasing interest in the role of selenium (Se) in the development of diabetes and have drawn some conclusions [8, 9]. Se is a vital trace element in biological processes and exerts its biological function through selenoproteins [8]. Notably, selenoproteins are believed to be involved in antioxidant response, immune system function, and regulation of thyroid hormones [8]. In recent years, the protective effect of Se on T2DM has been highlighted through the enhancement of internal antioxidant defense [10]. As a result, there has been a widespread promotion of increasing Se intake through diet [8]. However, based on the results of cross-sectional studies conducted in China [11, 12] and the United States [13–15], as well as prospective studies conducted in Italy [16, 17], there is an association between higher Se intake and an increased prevalence of T2DM. Furthermore, a previous meta-analysis [18] supports the above view, which is different from the theory that Se is beneficial to T2DM.

Therefore, our study aims to investigate the relationship between dietary Se intake and the prevalence of T2DM. Additionally, certain population subgroups may be more vulnerable to the impact of Se, but research in this area is limited. This research will control for variables and conduct stratified analysis based on age, gender, and thyroid autoimmunity, to provide advice for informed decisions regarding Se supplementation.

## Methods

### Study population

**Fig. 1:**
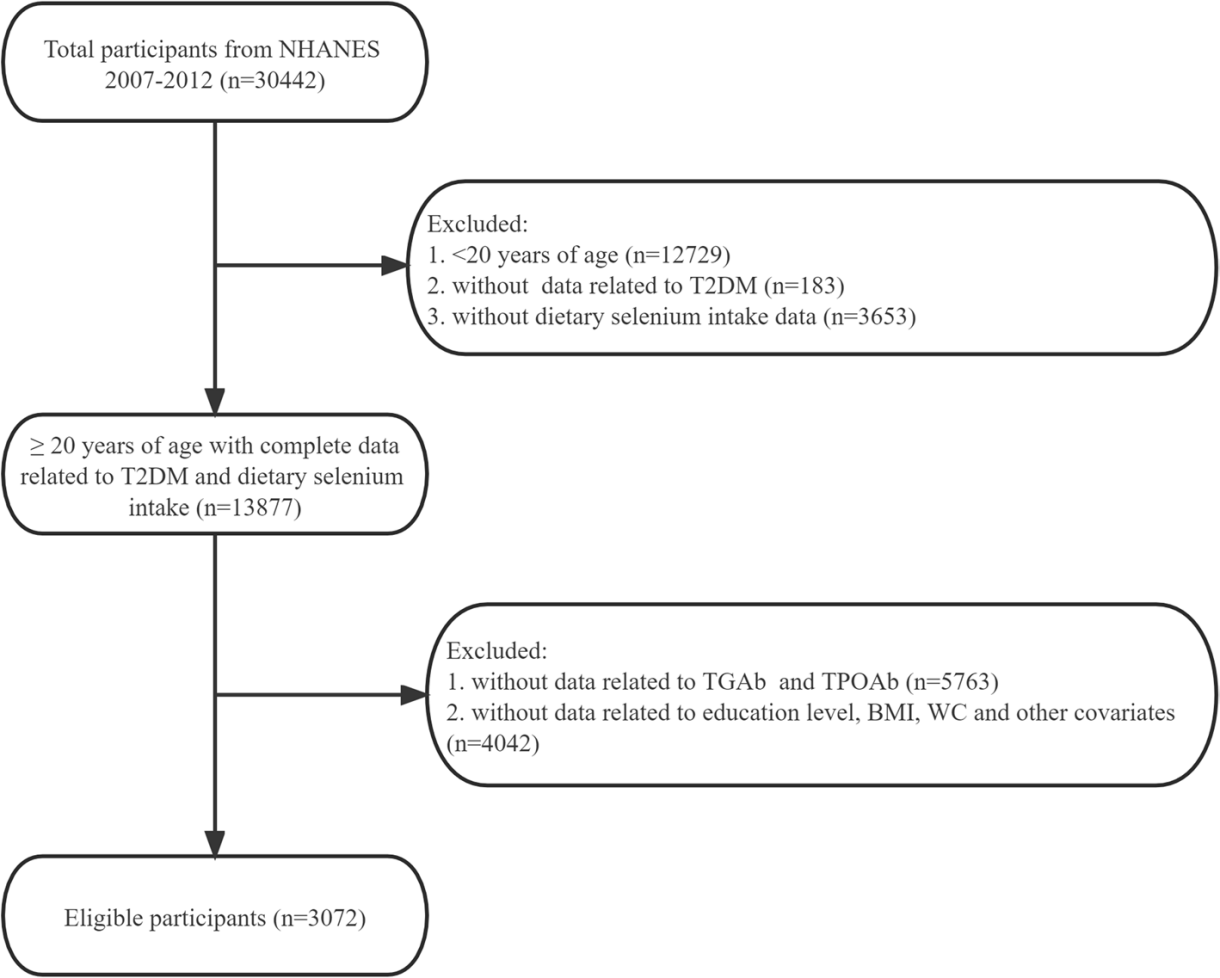
Flowchart of participants enrollment

This cross-sectional analysis enrolled individuals aged 20 years and above from the National Health and Nutrition Examination Survey (NHANES) conducted between 2007 and 2012. NHANES, carried out by the National Center for Health Statistics of the Centers for Disease Control and Prevention, is a comprehensive nationwide survey aimed at assessing the health and nutritional status of non-hospitalized residents in the United States. The survey methodology, including population and sample survey methods, is available in detail on the NHANES website. The NHANES survey protocol obtained ethical approval from the Ethics Review Committee of the National Center for Health Statistics, and all participants provided written informed consent. Between 2007 and 2012, a total of 30,442 participants were included in this study. The analysis specifically focused on individuals aged 20 years and above who had available data on the dietary intake of Se and T2DM. Participants with missing data on TGAb, TPOAb, and other covariates were excluded, resulting in a final sample size of 3,072 eligible participants for the analysis (Fig. 1).

### Diagnosis of T2DM

T2DM was the outcome variable, defined as: (1) ever been told by a doctor or health professional that you have diabetes or sugar diabetes? (2) HbA1c > 6.5%, (3) fasting glucose ≥ 7.0mmol/L, (4) random blood glucose ≥ 11.1mmol/L, (5) two-hours OGTT blood glucose ≥ 11.1mmol/L, (6) use of diabetes medication or insulin. Meeting any of the above criteria and excluding type 1 diabetes leads to the diagnosis of type 2 diabetes.

### Dietary Se intake assessment

The dietary intake of Se was evaluated through two interviewer-administered 24-h recalls. These recalls were conducted in person at the mobile examination center (MEC) and via telephone 3–10 days later. The dietary Se intake was calculated in micrograms per day (μg/day). The final dietary Se intake utilized in this study was obtained by averaging the two measurements of Se intake.

### Covariates

Participants completed a self-reported demographic questionnaire, which included inquiries about age, sex, race/ethnicity, and education level. Anthropometric measurements, such as body mass index (BMI) and waist circumference (WC), were also taken. The study also assessed various laboratory parameters, including 25OHD(D2 + D3), HDL-cholesterol, LDL-cholesterol, triglycerides, and total cholesterol. The dietary intake of energy, fiber, total fat, vitamin D(D2 + D3), magnesium, iron, zinc, and copper, alpha-carotene, beta-carotene, vitamin C, vitamin E was evaluated using the average of two interviewer-administered 24-h recalls. Smoking status was categorized as never smoker (having smoked fewer than 100 cigarettes in life), former smoker (having smoked more than 100 cigarettes in life but not currently smoking), and current smoker (having smoked more than 100 cigarettes in life and currently smoking some days or every day). Hypertension was defined as having an average systolic blood pressure ≥ 140mmHg or diastolic blood pressure ≥ 90mmHg. If only one reading was obtained, that reading was considered the average; if there were multiple readings, the first reading was always excluded. Cardiovascular disease (CVD) was defined as meeting any of the following criteria: (1) coronary heart disease, (2) congestive heart disease, (3) heart attack, (4) stroke, or (5) angina. Thyroid autoimmunity was categorized as negative (TGAb < 4.0IU/mL and TPOAb < 9.0 IU/mL) or positive (TGAb ≥ 4.0IU/mL or TPOAb ≥ 9.0 IU/mL).

### Statistical analysis

All data were represented as mean ± standard deviation (SD) for continuous variables and as percentages for categorical variables. To assess the continuous variables and categorical variables, chi-square tests (for categorical variables), t-tests (for variables with a normal distribution), and Kruskal–Wallis tests (for variables with a skewed distribution) were utilized.

The data on Se intake were log2-transformed due to its skewed distribution. Multivariate logistic regression modeling was used to estimate odds ratios (ORs) and 95% confidence intervals (CIs) for the risk of T2DM. Three sequential models were employed to control for potential confounders. The non-adjusted model did not include any adjustments. Model 1 was adjusted for age, sex, and race/ethnicity. Model 2 was adjusted for age, sex, race/ethnicity, education level, BMI, WC, 25OH(D2 + D3), HDL-Cholesterol, LDL-cholesterol, triglyceride, total cholesterol, energy, fiber, total fat, vitamin D(D2 + D3), magnesium, iron, zinc, copper, smoking status, hypertension, CVD, and thyroid autoimmunity.

Subgroup analyses were conducted based on age (categorized as binary), sex, and thyroid autoimmunity to assess the potential impact of these variables on the relationship. A smooth curve fit was applied to describe the nonlinear relationship between dietary Se intake and T2DM. After stratifying the analysis by age, sex, and thyroid autoimmunity, there appeared to be a threshold effect in both males and females. The two-segment linear regression model indicated a threshold effect in males with an inflection point of 90.51 μg and an inverted U-shaped relationship in females with an inflection point of 109.90 μg, respectively.

We further performed sensitivity analyses. To avoid losing more sample of subjects (*n* = 745) because of the missing data of the dietary intake of alpha-carotene, beta-carotene, vitamin C, vitamin E, we adjusted them after excluding the participants without data of dietary intake.

Statistical analyses were performed using the R® software package (v.4.2.0, http://www.r-project.org, accessed on 22 April 2022) and Empower® software (v.4.2, http://www.empowerstats.com, X&Y Solutions, Inc. Boston, MA, USA). A significance level of *p* < 0.05 (two-sided) was utilized to determine statistical significance.

## Results

### Baseline characteristic of participants

After excluding individuals with missing data, a total of 3072 participants were included in the final analysis. The baseline characteristic was displayed in Table 1. The average age of the participants was 50.74 years and the proportions of males and females were nearly equal (49.12% vs. 50.88%). The majority of participants were Non-Hispanic White and had attained education level beyond high school. Compared to non-T2DM participants, those with T2DM were characterized by older age and higher values of BMI, WC, and triglyceride levels. They also exhibited lower levels of 25OHD(D2 + D3), HDL-Cholesterol, LDL-Cholesterol, total cholesterol, and dietary intake, except for vitamin D (D2 + D3). Moreover, T2DM participants had higher proportions of hypertension, CVD, and positive thyroid autoimmunity and a lower proportion of now smoker. The baseline characteristic of participants of sensitivity analyses was displayed in Table S1.

**Table 1:** Baseline characteristic of participants

Covariates	Total	T2DM		*P*-value
		No	Yes	
*N*	3072	2416	656	
Age (years), Mean ± SD	50.74 ± 17.83	47.64 ± 17.60	62.17 ± 13.50	< 0.001
Sex, *n* (%)				0.055
Male	1509 (49.12%)	1165 (48.22%)	344 (52.44%)	
Female	1563 (50.88%)	1251 (51.78%)	312 (47.56%)	
Race/Ethnicity, *n* (%)				0.026
Non-Hispanic White	1491 (48.54%)	1186 (49.09%)	305 (46.49%)	
Non-Hispanic Black	581 (18.91%)	430 (17.80%)	151 (23.02%)	
Mexican American	476 (15.49%)	380 (15.73%)	96 (14.63%)	
Other Race	524 (17.06%)	420 (17.38%)	104 (15.85%)	
Education Level, *n* (%)				< 0.001
Less than high school	846 (27.54%)	604 (25.00%)	242 (36.89%)	
High school	710 (23.11%)	542 (22.43%)	168 (25.61%)	
More than high school	1516 (49.35%)	1270 (52.57%)	246 (37.50%)	
BMI (kg/m2), Mean ± SD	28.83 ± 6.39	27.97 ± 5.93	31.99 ± 6.99	< 0.001
WC (cm), Mean ± SD	99.10 ± 15.69	96.56 ± 14.81	108.46 ± 15.30	< 0.001
25OHD(D2 + D3) (nmol/L), Mean ± SD	63.33 ± 25.66	64.06 ± 25.96	60.64 ± 24.33	0.002
HDL-Cholesterol (mg/dL), Mean ± SD	53.66 ± 15.36	54.84 ± 15.60	49.34 ± 13.62	< 0.001
LDL-Cholesterol (mg/dL), Mean ± SD	115.92 ± 35.44	118.07 ± 34.56	108.00 ± 37.52	< 0.001
Triglyceride (mg/dL), Mean ± SD	125.20 ± 66.63	117.81 ± 62.78	152.42 ± 73.08	< 0.001
Total Cholesterol (mg/dL), Mean ± SD	194.63 ± 40.54	196.48 ± 39.72	187.84 ± 42.78	< 0.001
Dietary intake, Mean ± SD				
Energy (kcal)	1987.91 ± 798.52	2042.98 ± 805.82	1785.12 ± 736.91	< 0.001
Fiber (gm)	16.39 ± 8.62	16.56 ± 8.65	15.74 ± 8.45	0.029
Total fat (gm)	73.94 ± 36.08	75.27 ± 36.26	69.01 ± 34.99	< 0.001
Vitamin D(D2 + D3) (mcg)	4.47 ± 4.13	4.47 ± 4.17	4.45 ± 3.95	0.893
Magnesium (mg)	282.20 ± 117.13	287.62 ± 119.33	262.24 ± 106.38	< 0.001
Iron (mg)	14.81 ± 7.38	15.01 ± 7.45	14.07 ± 7.08	0.004
Zinc (mg)	11.16 ± 6.15	11.32 ± 6.36	10.56 ± 5.27	0.005
Copper (mg)	1.24 ± 0.60	1.26 ± 0.63	1.15 ± 0.48	< 0.001
Selenium (μg)	107.72 ± 49.24	109.13 ± 49.73	102.54 ± 47.06	0.002
Smoking status, *n* (%)				< 0.001
Never	1657 (53.94%)	1346 (55.71%)	311 (47.41%)	
Former	802 (26.11%)	564 (23.34%)	238 (36.28%)	
Now	613 (19.95%)	506 (20.94%)	107 (16.31%)	
Hypertension, *n* (%)				< 0.001
No	1779 (57.91%)	1591 (65.85%)	188 (28.66%)	
Yes	1293 (42.09%)	825 (34.15%)	468 (71.34%)	
CVD, *n* (%)				< 0.001
No	2719 (88.51%)	2226 (92.14%)	493 (75.15%)	
Yes	353 (11.49%)	190 (7.86%)	163 (24.85%)	
Thyroid autoimmunity, *n* (%)				0.046
Negative	2605 (84.80%)	2065 (85.47%)	540 (82.32%)	
Positive	467 (15.20%)	351 (14.53%)	116 (17.68%)	

*BMI* Body Mass Index, *WC* Waist Circumference, *CVD*, Cardiovascular disease

### Association of dietary Se intake and T2DM

**Fig. 2:**
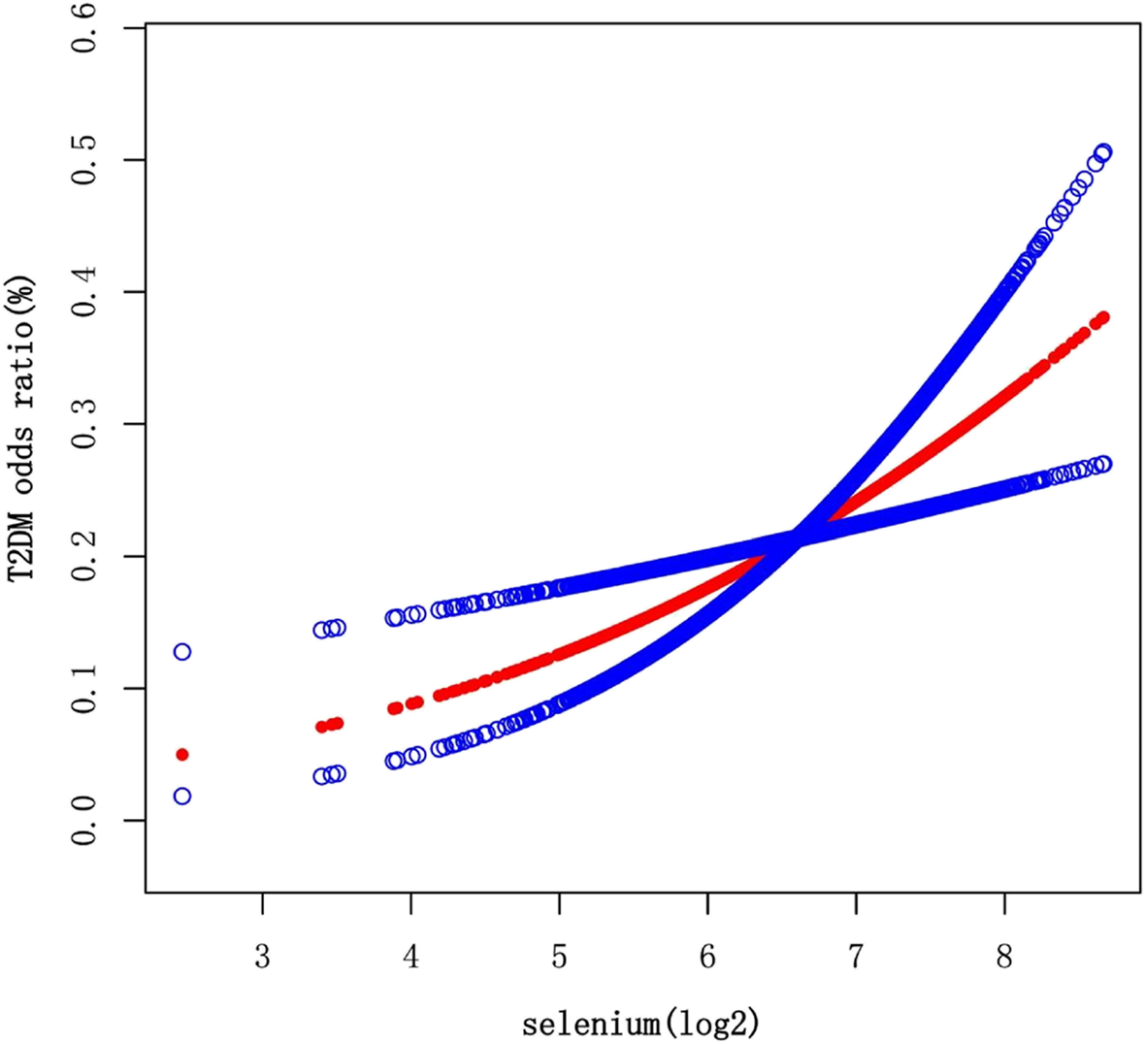
The association between Se intake and T2DM. Each red dot represents the dietary Se intake level, forming a continuous fitting curve. The area between the blue dashed lines is considered as 95% confidence interval. Age, sex, race/ethnicity, education level, BMI, WC, 25OHD(D2 + D3), HDL-Cholesterol, LDL-cholesterol, triglyceride, total cholesterol, energy, fiber, total fat, vitamin D (D2 + D3), magnesium, iron, zinc, copper, smoking status, hypertension, CVD, thyroid autoimmunity were adjusted

In the non-adjusted analysis (Table 2), a significant inverse association was found between log2-transformed Se intake and T2DM (OR = 0.83, 95% CI: 0.73, 0.94, *p* = 0.0039). When Se intake(log2-transformed) was divided into four equal groups, both Q2 and Q4 showed similar significant results. However, after adjusting for age, sex, and race/ethnicity (model 1), the association between Se intake(log2-transformed) and T2DM became positive but not statistically significant (OR = 1.09, 95% CI: 0.93, 1.26, *p* = 0.2870). In the fully adjusted model (model 2), a significant positive association between Se intake(log2-transformed) and T2DM was observed (OR = 1.49, 95% CI: 1.16, 1.90, *p* = 0.0017). However, when Se(log2-transformed) was divided into four equal groups for analysis, no significant association was found, with a *p*-value of 0.0739 for the trend test. A smooth curve fitting was conducted to illustrate the nonlinear relationship (Fig. 2).

**Table 2:** Association of dietary Se intake and T2DM

Exposure	Non-adjusted model	Model 1	Model 2
	OR (95% CI) *p* value	OR (95% CI)* p* value	OR (95% CI) p value
Selenium intake (log2-transformed)	0.83 (0.73, 0.94) 0.0039	1.09 (0.93, 1.26) 0.2870	1.49 (1.16, 1.90) 0.0017
Q1 (2.46–6.18)	1.00 (Reference	1.00 (Reference)	1.00 (Reference)
Q2 (6.18–6.65)	0.77 (0.61, 0.98) 0.0321	0.88 (0.68, 1.14) 0.3236	0.96 (0.72, 1.29) 0.7939
Q3 (6.65–7.06)	0.81 (0.64, 1.03) 0.0812	1.04 (0.80, 1.35) 0.7510	1.31 (0.94, 1.81) 0.1113
Q4 (7.06–8.67)	0.62 (0.49, 0.80) 0.0002	1.00 (0.75, 1.33) 0.9907	1.37 (0.89, 2.09) 0.1534
*p* for trend	0.0006	0.7294	0.0739

Non-adjusted model: adjusted for none

Model 1: age, sex, and race/ethnicity were adjusted

Model 2:age, sex, race/ethnicity, education level, BMI, WC, 25OHD(D2 + D3), HDL-Cholesterol, LDL-cholesterol, triglyceride, total cholesterol, energy, fiber, total fat, vitamin D(D2 + D3), magnesium, iron, zinc, copper, smoking status, hypertension, CVD, thyroid autoimmunity were adjusted

### Association of dietary Se intake and T2DM stratified by age, sex, and thyroid autoimmunity

**Fig. 3:**
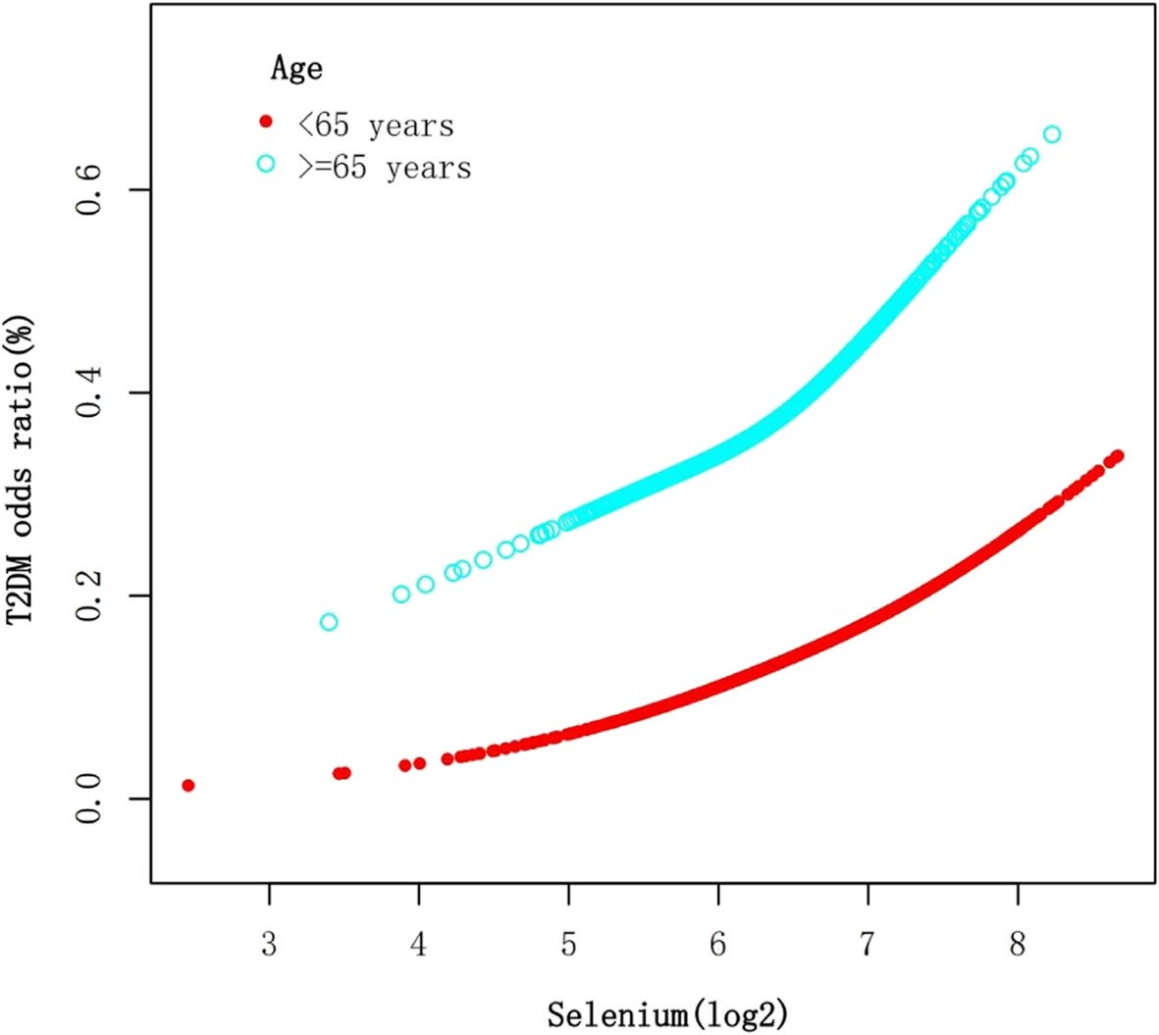
The association between Se intake and T2DM stratified by age. Each stratification adjusted for all the factors (age, sex, race/ethnicity, education level, BMI, WC, 25OHD(D2 + D3), HDL-Cholesterol, LDL-cholesterol, triglyceride, total cholesterol, energy, fiber, total fat, vitamin D (D2 + D3), magnesium, iron, zinc, copper, smoking status, hypertension, CVD, thyroid autoimmunity) except the stratification

**Fig. 4:**
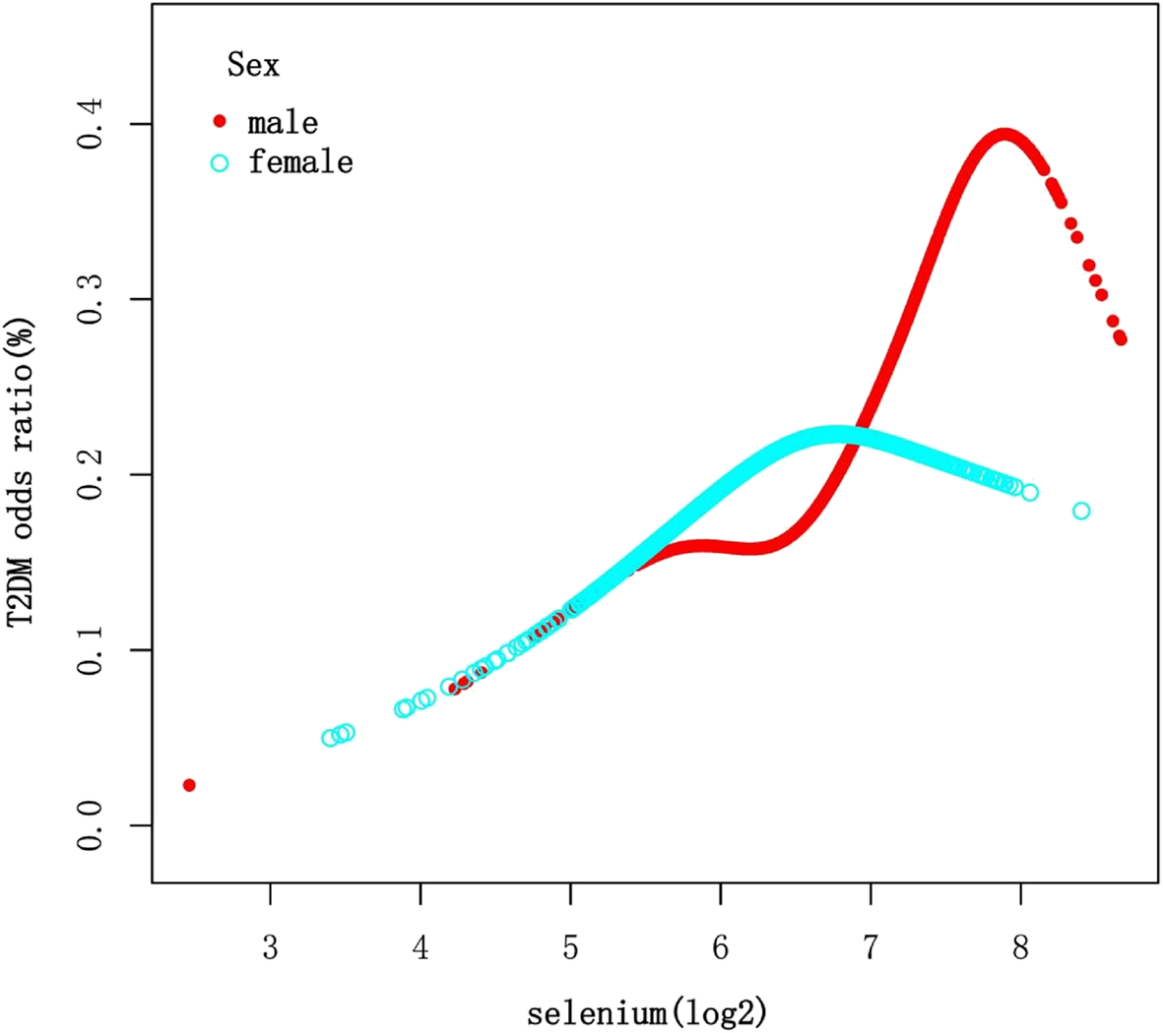
The association between Se intake and T2DM stratified by sex. Each stratification adjusted for all the factors (age, sex, race/ethnicity, education level, BMI, WC, 25OHD(D2 + D3), HDL-Cholesterol, LDL-cholesterol, triglyceride, total cholesterol, energy, fiber, total fat, vitamin D (D2 + D3), magnesium, iron, zinc, copper, smoking status, hypertension, CVD, thyroid autoimmunity) except the stratification

**Fig. 5:**
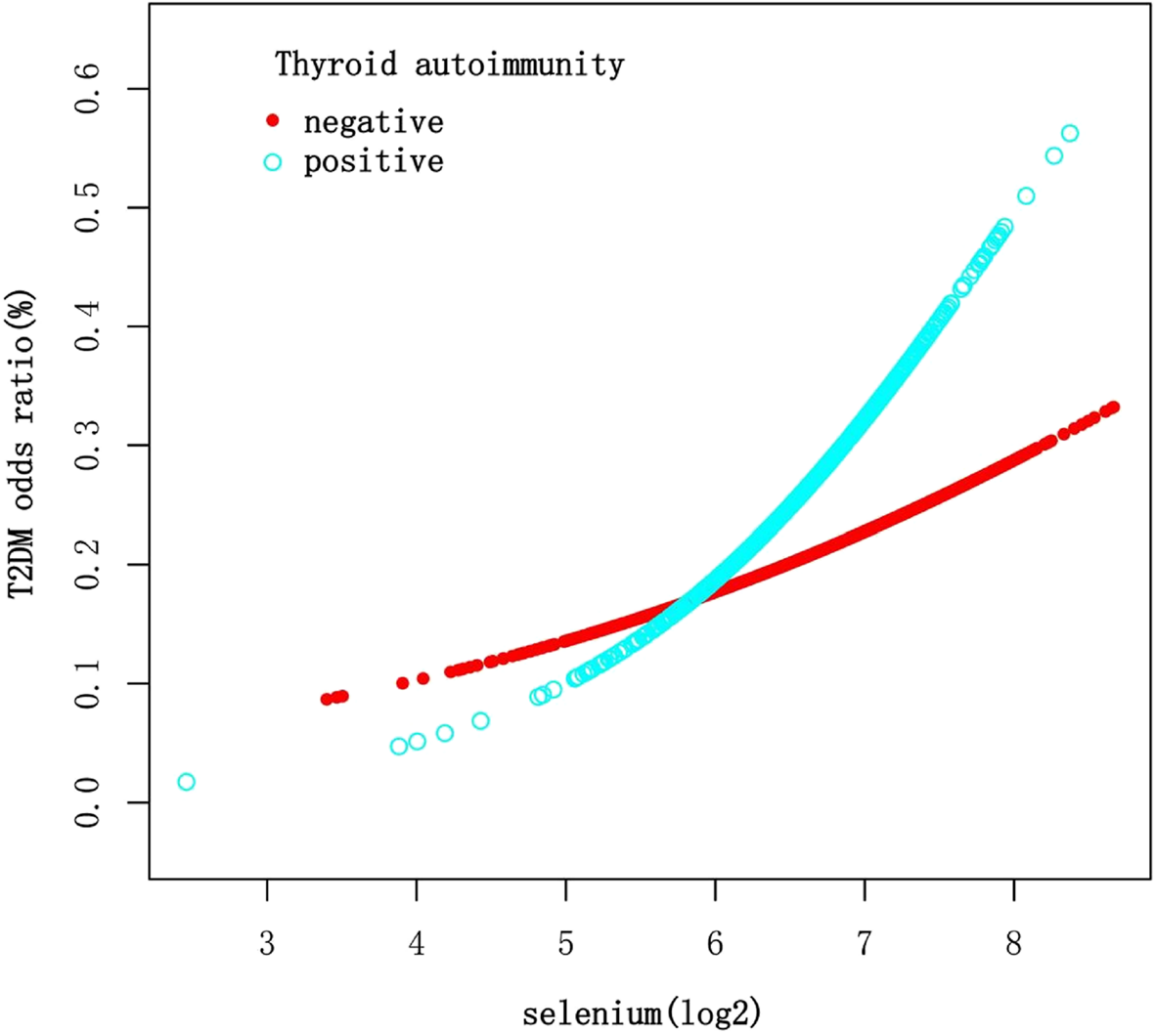
The association between Se intake and T2DM stratified by thyroid autoimmunity. Each stratification adjusted for all the factors (age, sex, race/ethnicity, education level, BMI, WC, 25OHD(D2 + D3), HDL-Cholesterol, LDL-cholesterol, triglyceride, total cholesterol, energy, fiber, total fat, vitamin D (D2 + D3), magnesium, iron, zinc, copper, smoking status, hypertension, CVD, thyroid autoimmunity) except the stratification

After stratifying the data by age, sex, and thyroid autoimmunity, a significant positive association between Se intake (log2-transformed) and T2DM was observed in individuals under 65 years of age, males, and those with negative thyroid autoimmunity (Table 3). Smooth curve fittings (Figs. 3, 4, 5) were conducted to illustrate the linear or nonlinear relationship. A two-segment linear regression model was analyzed for sex stratification, revealing a threshold effect in males with an inflection point of 6.5 (90.51 μg), and an inverted U-shaped relationship in females with an inflection point of 6.78 (109.90 μg), respectively (Table 4). When Se intake exceeded 90.51 μg in males and was below 109.90 μg in females, a consistently significant positive association with T2DM was observed. However, when Se intake was below 90.51 μg in males, the positive relationship was not significant. Additionally, when Se intake exceeded 109.90 μg in females, an inverse association with T2DM was observed, but it was not statistically significant.

**Table 3:** Association of dietary Se intake and T2DM stratified by sex, age, and thyroid autoimmunity

Exposure	*N*	OR (95% CI) *p* value
Selenium intake (log2-transformed)	3072	1.49 (1.16, 1.90) 0.0017
Stratified by age		
< 65 years	2250	1.97 (1.04, 3.73) 0.0370
≥ 65 years	822	0.97 (0.44, 2.13) 0.9398
Stratified by sex		
male	1509	1.77 (1.24, 2.52) 0.0018
female	1563	1.28 (0.89, 1.85) 0.1825
Stratified by thyroid autoimmunity		
negative	2605	1.43 (1.09, 1.89) 0.0098
positive	467	1.55 (0.83, 2.89) 0.1681

Adjusted for age, sex, race/ethnicity, education level, BMI, WC, 25OHD(D2 + D3), HDL-Cholesterol, LDL-cholesterol, triglyceride, total cholesterol, energy, fiber, total fat, vitamin D(D2 + D3), magnesium, iron, zinc, copper, smoking status, hypertension, CVD, thyroid autoimmunity except the stratification

**Table 4:** Threshold effect analysis of Se intake on T2DM using two-piecewise linear regression

T2DM	OR (95%CI) *p* value
Male	
Inflection point	6.5
< 6.5	1.17 (0.72, 1.92) 0.5201
> 6.5	2.73 (1.60, 4.64) 0.0002
Log likelihood ratio	0.030
Female	
Inflection point	6.78
< 6.78	1.59 (1.06, 2.40) 0.0266
> 6.78	0.31 (0.09, 1.02) 0.0546
Log likelihood ratio	0.009

Adjusted for age, race/ethnicity, education level, BMI, WC, 25OHD(D2 + D3), HDL-Cholesterol, LDL-cholesterol, triglyceride, total cholesterol, energy, fiber, total fat, vitamin D(D2 + D3), magnesium, iron, zinc, copper, smoking status, hypertension, CVD, thyroid autoimmunity

### Sensitivity analyses

The sensitive analyses (Table S2) showed that the association between Se intake(log2-transformed) and T2DM became more strongly positive, although the p-value was statistically non-significant (OR = 1.75, 95% CI: 0.99, 3.09, *p* = 0.0548). However, in the subgroup analyses (Table S3), the above association still remain significantly positive in below 65 years of age and participants with negative thyroid autoimmunity. The association was not significant in neither males group nor females group.

## Discussion

The present study revealed a significant positive correlation between dietary Se intake and the risk of T2DM. Specifically, higher dietary Se intake is associated with an increased T2DM risk, particularly among individuals under 65 years of age, males, and those with negative thyroid autoimmunity. Notably, a threshold effect is observed in the gender stratification analysis. Our findings suggest that Se intake above 90.51 μg per day significantly raises the risk of T2DM in males, while intake below 109.90 μg per day significantly increases the risk in females.

The overall findings of this study align with several previous studies that have reported a positive correlation between blood Se concentrations and the incidence of T2DM [12, 19, 20]. Se, being a trace mineral element with a narrow therapeutic range and significant inter-individual variability [21], is thought to play a role in T2DM, a chronic metabolic disorder typically characterized by pancreatic β-cell dysfunction and insulin resistance [22]. Although the exact mechanism by which Se increases the risk of T2DM has not been fully elucidated, several studies have investigated potential underlying mechanisms. Satyanarayana S et al. have suggested that a diet high in Se may stimulate glucagon release, leading to hyperglycemia [23]. Additionally, an animal experiment has indicated that high Se levels can induce hepatic insulin resistance by either promoting lipolysis-induced damage from reactive oxygen species (ROS) or suppressing insulin-stimulated ROS signaling [24].

According to currently available epidemiologic studies suggesting that the relationship between Se and T2DM is controversial, which means that lower Se intake and higher Se intake may both be risk factors for T2DM [8, 25, 26]. This may be due to differences in baseline serum Se levels. It has been suggested that participants with lower Se status may benefit from Se supplementation, while high dietary Se supplementation is not recommended for those with already high serum or plasma Se concentrations of 122 μg/L or higher [8].

In our study, we observed that Se intake above 90.51 μg significantly increased the risk of T2DM in males, while selenium intake below 109.90 μg significantly increased the risk of T2DM in females. It is worth noting that the average (standard deviation) serum Se concentration in US residents, as measured in the 2003–2004 NHANES, was 137 (20)μg/L. [27]. As previously mentioned, the high baseline Se levels in the US population may contribute to the observed association between increased dietary Se intake and an increased risk of T2DM. Interestingly, sex hormones seem to have a direct influence on Se distribution and metabolism [28]. Earlier studies have reported a positive correlation between plasma Se levels, glutathione peroxidase (GPX) activity, and estrogen fluctuations in pre-menopausal women, suggesting a potential direct effect of estrogen on Se status [29]. In addition, there is a significant demand for Se in the testicles, resulting in a preferential supply of Se to the testicles in males [30]. This means that, even with the same Se intake, the increase in serum Se levels attributed to this specific intake may not be equal between men and women. This observation could potentially explain why a lower Se intake is associated with a significantly increased risk of diabetes in women, while a higher Se intake is associated with a significantly increased risk of diabetes in men.

Our study also confirmed that the positive association between dietary Se intake and the risk of T2DM remained significant in individuals with negative thyroid autoimmunity. Several clinical studies have demonstrated that Se supplementation reduces TPOAb and/or TGAb titers and improves thyroid function in patients with Hashimoto's thyroiditis (positive for TGAb and TPO-Ab), suggesting that Se may be beneficial in treating thyroid autoimmunity [31–33]. However, some current studies have suggested a potential increased risk of diabetes with the use of Se supplements in adults. In our study, we found that Se intake was not significantly associated with the risk of T2DM in the population with positive thyroid autoimmunity. This implies that individuals with positive thyroid autoimmunity may not need to be concerned about an increased risk of T2DM when increasing Se intake to treat thyroid autoimmunity.

Our study has several strengths. Firstly, we utilized a large sample size and adjusted for a greater number of confounding variables, which enhanced the reliability of our results. Secondly, the data used in our study were obtained from NHANES, a survey known for its rigorous sampling design, high-quality data collection, and meticulous quality control procedures. This ensures the robustness and validity of our findings. Finally, our study employed fitted smoothed curves and conducted threshold effects analysis to examine the nonlinear relationship between dietary selenium intake and the risk of T2DM. However, it is important to consider several potential limitations of this study. Firstly, due to the cross-sectional nature of the study design, we were only able to observe an association between Se intake and the prevalence of T2DM, and cannot establish a direct causal relationship. Secondly, the study population selected for this research had relatively high Se exposure and demonstrated elevated serum Se concentrations, partly attributed to the Se-rich content of U.S. soils [27, 34]. As a result, it may not be appropriate to generalize the conclusions to other populations with different Se levels. Because the Se content of foods varies widely depending on soil conditions, dietary assessments of Se intake are usually less accurate than measurements of serum Se concentrations. Lastly, the estimation of dietary Se intake relied on participant recall, which introduces the possibility of recall bias.

Except for weight loss, increasing physical activity, adopting a healthy diet also remains one of the first-line strategies for the management of T2DM. Altogether, our results suggest that higher selenium intake might be a new predictor for the presence of T2DM and should be validated in future large prospective studies in different populations. The identification of different threshold effects in males and females provides valuable insights for future research and the development of targeted interventions for T2DM prevention. In addition, this study investigated the relationship between selenium and thyroid autoimmune stratified T2DM. The study provides some research contributions to whether selenium supplementation therapy for thyroid autoimmunity may also increase the risk of type 2 diabetes.

## Conclusions

The present study found a positive relationship between Se intake and the risk of T2DM. This association is particularly significant in younger individuals, males, and those with negative thyroid autoimmunity. It is important to replicate our findings in future studies conducted on diverse populations, and further establish cohort studies to investigate the potential causal relationship between selenium intake and the occurrence of T2DM.

### Supplementary Information

**Supplementary Material 1.**

**Supplementary Material 2.**

**Supplementary Material 3.**

## Data Availability

The NHANES data sets can be accessed by the public through the Centers for Disease Control and Prevention website at https://wwwn.cdc.gov/nchs/nhanes/Default.aspx.
